# Secondhand Smoke Exposure Causes Bronchial Hyperreactivity *via* Transcriptionally Upregulated Endothelin and 5-hydroxytryptamine 2A Receptors

**DOI:** 10.1371/journal.pone.0044170

**Published:** 2012-08-27

**Authors:** Lei Cao, Yaping Zhang, Yong-Xiao Cao, Lars Edvinsson, Cang-Bao Xu

**Affiliations:** 1 Division of Experimental Vascular Research, Institute of Clinical Science in Lund, Lund University, Lund, Sweden; 2 Department of Pharmacology, Xi'an Jiaotong University College of Medicine, Xi'an, Shaanxi, People's Republic of China; University Hospital Freiburg, Germany

## Abstract

**Background:**

Cigarette smoke exposure is strongly associated with airway hyperreactivity (AHR) which is the main characteristic seen in asthma. The intracellular MAPK signaling pathways are suggested to be associated with the airway damage to the AHR. In the present study, we hypothesize that secondhand cigarette smoke (SHS) exposure upregulates the bronchial contractile receptors *via* activation of the Raf/ERK/MAPK pathway.

**Methodology/Principal Findings:**

Rats were exposed to SHS for 3 h daily for up to 8 weeks. The receptor agonists-induced bronchial contractile reactivity was analyzed with a sensitive myograph system. The mRNA transcription and protein translation of the target receptors and the kinases in Raf/ERK/MAPK pathway were investigated by real-time PCR, Western blotting and immunofluorescence, respectively. Compared with exposure to fresh air, SHS induced enhanced bronchial contractile responses mediated by the 5-hydroxytryptamine 2A (5-HT_2A_) receptors as well as the endothelin type B (ET_B_) and type A (ET_A_) receptors. The response curves were shifted toward the left with an increased maximal contraction (E_max_) demonstrating that SHS induced AHR. Additionally, the mRNA and protein levels of the 5-HT_2A_, ET_B_ and ET_A_ receptors were increased. Furthermore, SHS exposure increased the phosphorylation of Raf-1 and ERK1/2, but it did not alter p38 or JNK. A Raf-1 inhibitor (GW5074) suppressed the SHS-induced increase in the expression of 5-HT_2A_ and ET_A_ receptors and the receptor-mediated AHR.

**Conclusions/Significance:**

Our findings show that SHS exposure induces transcriptional upregulation of the 5-HT_2A_, ET_B_ and ET_A_ receptors in rat bronchial smooth muscle cells, which mediates AHR. The Raf/ERK/MAPK pathway is involved in SHS-associated receptor upregulation and AHR.

## Introduction

The inhalation of tobacco smoke, either *via* direct smoking or passive exposure, is a strong risk factor for the development of airway hyperreactivity (AHR) with increased respiratory symptoms [1]. Cigarette smoke has been noted in numerous studies to influence the development and/or the exacerbation of asthma [2]. Passive smoking, also known as secondhand smoke (SHS) exposure, is one of the main contributing factors during the early stage of AHR, which is a hallmark of asthma [3]. SHS constitutes a serious public health risk because the smoke emitted from the tip of a cigarette contains high concentrations of nicotine, carbon monoxide and many carcinogens [4]. However, there is still limited knowledge about the underlying mechanisms within the bronchial walls that account for the relationship between SHS exposure and AHR.

We have reported that SHS exposure induces tracheal hyperresponsiveness to receptor agonists of carbachol and endothelin-1 (ET-1) in an *in vivo* mouse model [5]. Accumulating evidence has revealed that some G-protein coupled receptors (GPCR) in bronchioles display plasticity that allows them to adapt to environmental changes. In the respiratory system, it is in particular the receptors that mediate contraction of airway smooth muscles with consequences for control of the bronchial lumen diameter and thus pulmonary ventilation [6]. The bronchioles are the major site of airway reactivity. Thus, the bronchial hyperreactivity is the key component and structure of AHR [7]. The present study focuses on rat intrapulmonary bronchi which are considered to be the primary site of AHR in airway disease. Previous *in vitro* studies using an organ culture model showed that exposure of isolated bronchi to dimethylsulfoxide-soluble smoking particles (DSP) altered airway endothelin [8] and thromboxane receptor expression [9]. Therefore, we hypothesized that SHS, a major risk factor in a number of airway diseases, may upregulate contractile receptors in the bronchi, which could subsequently be involved in the pathogenesis of AHR.

Because DSP have been shown to activate extracellular signal-regulated protein kinase 1 and 2 (ERK1/2) signaling [10], we hypothesize that there is a strong association between the activation of mitogen-activated protein kinase (MAPK)-mediated signal transduction and the transcriptional upregulation of GPCRs in the bronchi [6], [11]. To test this *in vivo*, we exposed rats for 3 h per day up to 8 weeks and examined the expression of several contractile receptors in bronchioles. In particular, we sought to address whether activation of the Raf/ERK/MAPK pathway is involved in SHS-induced bronchial hyperreactivity by direct analysis of the pathway phosphorylation with Western blot, and by examining effect of GW5074, a specific inhibitor of the pathway.

## Results

### The Effect of SHS Exposure on Bronchial Contraction Induced by Receptor Agonists

There was no significant difference in the bronchial contractile responses of animal exposed to SHS for 2 or 4 weeks compared to those exposed to fresh air (data not shown). Therefore, we only present results of 8 weeks exposure.

**Figure 1:**
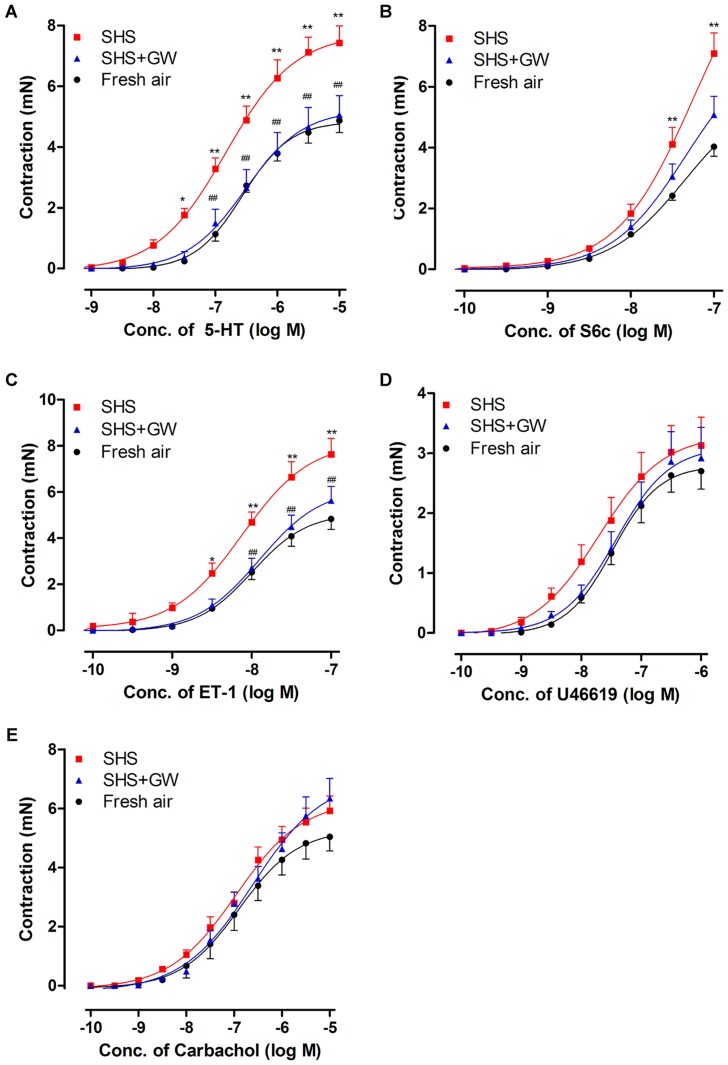
Contractile responses to the receptor agonists. The bronchial segments were from rats that were exposed to fresh air, SHS and SHS + GW5074. The response curves were induced by cumulative application of 5-HT (A), S6c (B), ET-1 (C), U46619 (D) and carbachol (E), respectively. The contractions are shown in absolute mN. The values are expressed as the mean ± SEM (*n* = 8–10). The statistical analysis was performed using two-way ANOVA with Bonferroni's post-test. ^*^P<0.05, ^**^ P<0.01 vs. fresh air exposure group; *^##^ P*<0.01 vs. SHS exposure group.

Cumulative administration of 5-hydroxytryptamine (5-HT), a general 5-HT receptor agonist, elicited a contractile response in the bronchial segments in a concentration-dependent manner (Fig. 1A). The contractility of the bronchial segment from animals that were exposed to SHS for 8 weeks was significantly enhanced, and the concentration-effect curve was shifted toward the left with an increase in E_max_ (7.73±0.56 mN, table 1) and an increase in pEC_50_ (6.81±0.11), compared to the fresh air group (E_max_: 4.84±0.35 mN, *P*<0.01; pEC_50_: 6.35±0.08, *P*<0.05).

**Table 1:** E_max_ and pEC_50_ values of contractile responses induced by the receptor agonists.

	Fresh air (*n* = 10)		SHS (*n* = 9)		SHS + GW5074 (*n* = 8)	
	E_max_	pEC_50_	E_max_	pEC_50_	E_max_	pEC_50_
5-HT	4.84±0.35	6.35±0.08	7.73±0.56^**^	6.81±0.11^*^	5.22±0.63^#^	6.45±0.13
S6c	4.25±0.31	7.44±0.10	7.82±0.68^**^	7.51±0.12	6.23±0.61	7.55±0.09
ET-1	5.10±0.36	8.06±0.13	8.18±0.69^**^	8.13±0.15	6.18±0.52^#^	7.97±0.07
U46619	2.80±0.24	7.46±0.08	3.31±0.41	7.65±0.14	3.18±0.46	7.44±0.11
carbachol	5.28±0.31	6.82±0.09	6.27±0.52	6.94±0.10	7.01±0.64	6.68±0.12

The bronchial segments were isolated from rats that were exposed to fresh air, SHS and SHS + GW5074 (0.5 mg/kg) for 8 weeks. The E_max_ values represent maximal contraction, the pEC_50_ values represent negative logarithm of the concentration that produces 50% of the maximal contractile effect. The E_max_ and pEC_50_ values from airway contractions were induced by 5-HT, S6c, ET-1, U46619 and carbachol. The values are expressed as the means ± SEM and *n* refers to the number of rats. The statistical analysis was performed using unpaired student's *t*-test with Welch's correction. ^*^P<0.05, ^**^P<0.01 vs. fresh air exposure group; *^#^P*<0.05 vs. SHS exposure group.

Sarafotoxin 6c (S6c), a specific ET_B_ receptor agonist, and ET-1, an activator of both ET_A_ and ET_B_ receptors, were used to study the ET receptor responses [12]. S6c was primarily used to produce concentration-contraction curves for the ET_B_ receptors in bronchial segments (Fig. 1B). Compared with the fresh air group, 8 weeks of SHS exposure resulted in a marked increase in contraction; E_max_ from 4.25±0.31 mN (fresh air, table 1) to 7.82±0.68 mN (SHS exposure, *P*<0.01).

As described in the methods section, the contractile responses caused by ET-1 were studied after desensitization of the ET_B_ receptors [8]. ET-1 induced a potent and sustained constriction in fresh bronchial segments (Fig. 1C) and this reaction was mediated by ET_A_ receptors. SHS exposure shifted the ET-1-induced contractile curve toward the left in a non-parallel manner with a significantly increased E_max_ (8.18±0.69 mN), compared to the fresh air group (5.10±0.36 mN, *P*<0.01).

U46619, a stable synthetic analog of thromboxane that activates thromboxane A_2_ receptors (TP receptors) induced contractile response in bronchi. There was no significant difference observed in the curves between the fresh air and the SHS exposure groups (Fig. 1D) when U46619 was applied. Furthermore, table 1 shows that SHS did not alter the E_max_ and pEC_50_. This result indicated that SHS exposure does not affect TP receptor-mediated contraction.

The muscarinic acetylcholine receptor agonist carbachol caused a concentration-dependent contraction in the bronchial segments of the rats exposed to fresh air (Fig. 1E). However, 8 weeks of SHS exposure did not alter the shape of the response curves in the fresh air groups. The E_max_ and pEC_50_ values were unchanged between the SHS and the fresh air groups (table 1).

### The Effect of SHS Exposure on Receptor mRNA Levels

**Figure 2:**
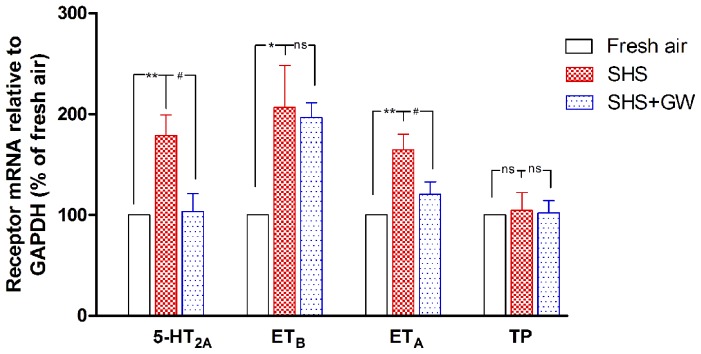
The levels of contractile receptor mRNA expression. The bronchial segments were from rats that were exposed to fresh air, SHS and SHS + GW5074. The receptor (5-HT_2A_, ET_B_, ET_A_ and TP) mRNA level is presented relative to that of the housekeeping gene GAPDH. The values are expressed as the mean ± SEM (n = 5–8). The statistical analysis was performed using one-way ANOVA with Dunnett's post-test. ^*^P<0.05, ^**^P<0.01 vs. fresh air exposure group; *^#^P*<0.05 vs. SHS exposure group; ns, not significant.

Real-time PCR analysis of total RNA extracted from the bronchial segments of all rats demonstrated the presence of mRNA coding for the 5-HT_2A_, ET_B,_ ET_A_ and TP receptors. In the fresh air group, the mRNA levels of the 5-HT_2A_, ET_B_ and ET_A_ receptors relative to the amount of GAPDH mRNA were 0.011±0.002, 0.003±0.002 and 0.026±0.004, respectively. After exposure to SHS for 8 weeks, the levels of the receptor mRNAs in the bronchial segments were 1.7 (5-HT_2A_), 2.1 (ET_B_) and 1.6 (ET_A_) times the levels seen in the control group (Fig. 2). This result showed that SHS exposure increased the mRNA levels of the 5-HT_2A_, ET_B_ and ET_A_ receptors. For the TP receptors, there was no significant difference in the mRNA levels between the fresh air and SHS groups.

### The Effect of SHS Exposure on Receptor Protein Expression

**Figure 3:**
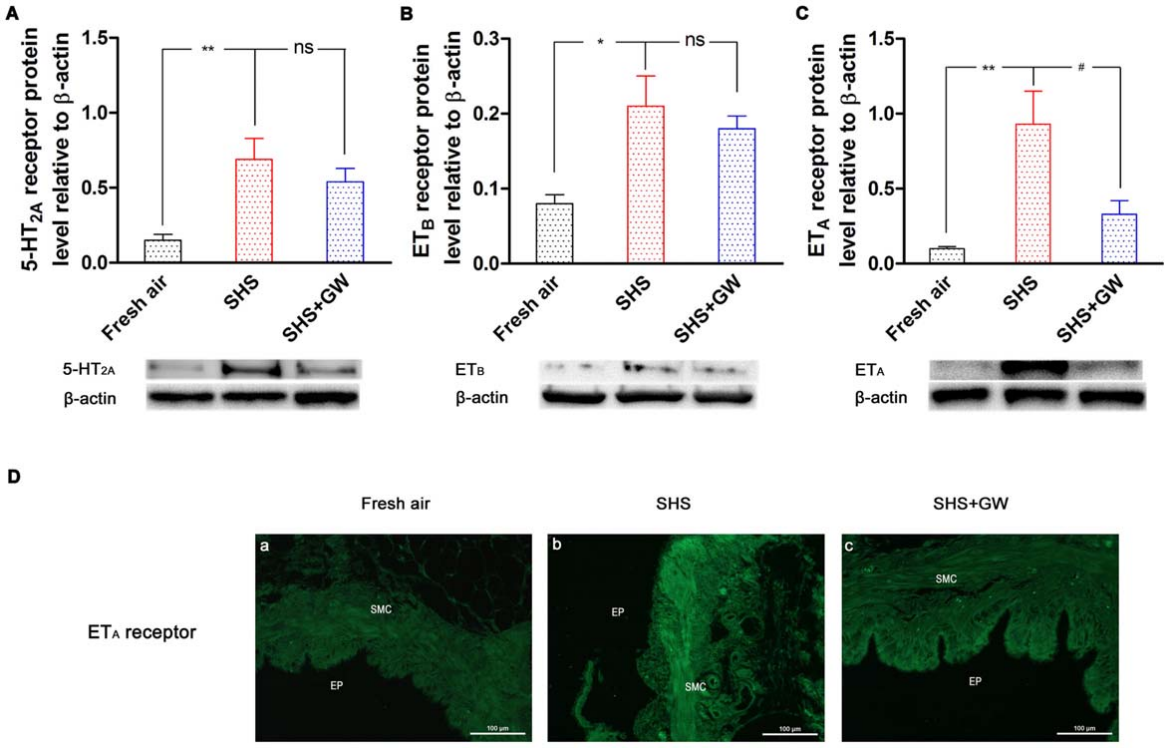
The levels of contractile receptor protein expression. The bronchial segments were from rats that were exposed to fresh air, SHS and SHS + GW5074. 5–HT_2A_ (A), ET_B_ (B) and ET_A_ (C) receptor protein levels were determined by Western blotting and presented relative to the level of β-actin. The values are expressed as the mean ± SEM (*n* = 5–8). The statistical analysis was performed using one-way ANOVA with Dunnett's post-test. ^*^P<0.05, ^**^P<0.01 vs. fresh air exposure group; *^#^P*<0.05 vs. SHS exposure group; ns, not significance. Immunofluorescence of ET_A_ receptors from rat bronchi sections is shown (D). The tissue was obtained from rats that were exposed to fresh air (a), SHS (b) and SHS + GW5074 (c). The sections were stained with FITC (green) to detect the ET_A_ receptor protein. SM: smooth muscle layer; EP: epithelium layer. Scale bar 100 μm.

To further analyze the changes in receptor expression after SHS exposure, quantitative protein analysis by Western blotting was performed. In the fresh air group, the protein levels of the receptors relative to the amount of β-actin were 0.15±0.04 (5-HT_2A_), 0.08±0.01 (ET_B_) and 0.10±0.01 (ET_A_). After exposure to SHS for 8 weeks the receptor protein levels were significantly elevated to 0.69±0.14 for 5-HT_2A_ (*P*<0.01; Fig. 3A), 0.21±0.04 for ET_B_ (*P*<0.05; Fig. 3B), and 0.93±0.22 for ET_A_ (*P*<0.01; Fig. 3C), respectively. These data are in agreement with the results observed for the function and mRNA studies.

To provide further evidence of the effect of SHS exposure on receptor protein expression levels, the ET_A_ receptor was chosen for immunofluorescence experiments because the Raf-1 inhibitor GW5074 only has inhibitory action on ET_A_ receptor regulation when we use Western blotting method. In the negative controls, the ET_A_ receptor protein was not expressed in the sections. In the fresh air group, the ET_A_ receptor antibodies exhibited weak immunoreactivity in the smooth muscle cells (SMC) (Fig. 3D [a]). After 8 weeks of SHS exposure, the bronchial segments showed increased immunoreactivity in the SMC layer (Fig. 3D [b]). However, there was no significant increase in ET_A_ receptor protein expression in the epithelium. The immunofluorescence results for the ET_A_ receptor are consistent with the Western blotting results.

### The Effect of SHS Exposure on Raf/ERK/MAPK Activation

**Figure 4:**
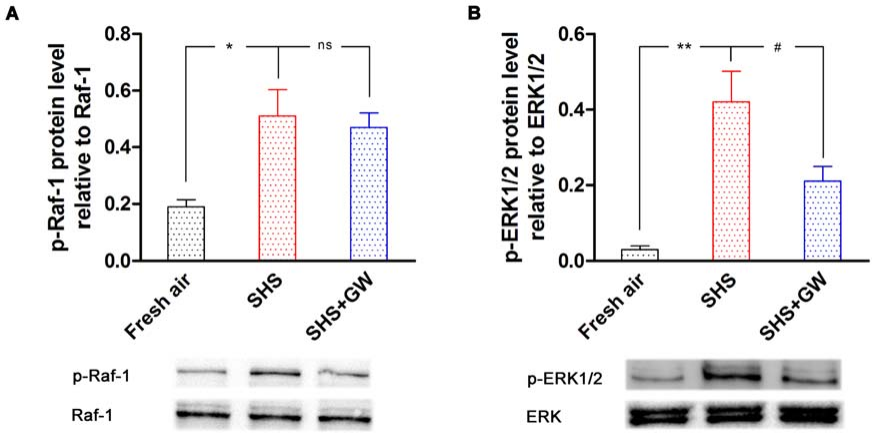
The activation (phosphorylation) of Raf/ERK/MAPK signaling pathway proteins. The bronchial segments were from rats that were exposed to fresh air, SHS and SHS + GW5074. The phosphorylated (p)-Raf-1 (A) and p-ERK1/2 (B) protein levels are presented relative to the total Raf-1 or total ERK1/2 level, respectively. The values are expressed as the mean ± SEM (*n* = 5–6). The statistical analysis was performed using one-way ANOVA with Dunnett's post-test. ^*^P<0.05, ^**^P<0.01 vs. fresh air exposure group; *^#^P*<0.05 vs. SHS exposure group; ns, not significance.

To explore the relationship between SHS exposure and the Raf/ERK/MAPK signaling pathway, we examined if the MAPKs were activated following SHS. Thus, the phosphorylation of Raf-1, ERK1/2, p38 and JNK were analyzed by Western blotting. The results showed that the protein levels of phosphorylated (p)-Raf-1 and p-ERK1/2 in fresh air exposed rats were 0.19±0.02 and 0.08±0.01 relative to Raf-1 or ERK1/2, respectively. After 8 weeks of SHS exposure, the phosphorylation levels of protein were increased to 0.51±0.09 (p-Raf-1, *P*<0.05; Fig. 4A) and 0.42±0.08 (p-ERK1/2, *P*<0.01; Fig. 4B), respectively. This finding demonstrated that SHS exposure increased the phosphorylation of Raf and ERK1/2. In contrast, the protein levels of p-JNK and p-p38 in SHS-exposed bronchi were not significantly different compared to those in fresh bronchi (data not shown). The results suggest that the Raf/ERK1/2 pathway is the main signal transmission mechanism involved in the bronchial alterations induced by SHS exposure.

### The Effect of GW5074

In order to further elucidate the involvement of the Raf/ERK/MAPK signaling pathway in SHS, we examined the effect of the Raf inhibitor GW5074 (3-[3, 5-dibromo-4- hydroxybenzylidine]-5-iodo-1, 3-dihydro-indol-2-one) on SHS-induced changes in bronchioles. The results showed that GW5074 inhibited the SHS-induced receptor upregulation. In our investigation of the effects of SHS on receptor function, treatment with GW5074 attenuated the SHS-induced enhanced bronchial contractions induced by 5-HT (Fig. 1A) and ET-1 (Fig. 1C). The E_max_ values of the concentration-contraction curves induced by 5-HT and ET-1 in the inhibitor group were significantly lower than those found in the group that received SHS only (table 1). GW5074 did not significantly alter the E_max_ values of S6c, U46619 or the carbachol-induced bronchial concentration-response curves.

Consistent with the results for contraction, the mRNA levels of the 5-HT_2A_ and ET_A_ receptors in the GW5074 treatment group were reduced compared with those in the SHS-exposed group (*P*<0.05; Fig. 2). The ET_B_ receptor mRNA (Fig. 2) and protein (Fig. 3B) were unaltered after inhibition. GW5074 did not affect TP receptor mRNA levels (Fig. 2). The Raf-1 inhibitor decreased the protein level of ET_A_ receptors after SHS exposure (*P*<0.05; Fig. 3C) and the protein expression of 5-HT_2A_ displayed a decreasing trend (*P*>0.05; Fig. 3A). Based on immunostaining, the bronchial segments from SHS-exposed animals treated with GW5074 showed decreased ET_A_ receptor protein expression (Fig. 3D[c]) in the smooth muscle layer in comparison with the SHS-exposed animals that were not treated with inhibitor.

Finally, we studied the effects of GW5074 on the activation of the Raf/ERK/MAPK signaling pathway in response to SHS exposure. The results showed that Raf-1 inhibitor GW5074 did not alter SHS-induced elevation of the p-Raf-1 level in the bronchi (Fig. 4A). However, p-ERK1/2 protein levels in the GW5074 treatment group were lower than in the SHS-exposed group (*P*<0.05; Fig. 4B), indicating that GW5074 reduced phosphorylation of ERK1/2. This finding suggests that the Raf/ERK/MAPK pathway is activated by SHS exposure and is associated with SHS-induced changes in bronchial receptor expression.

## Discussion

This is the first *in vivo* study to demonstrate that SHS exposure induces transcriptional upregulation of bronchial 5-HT_2A_, ET_B_ and ET_A_ receptors, which is mediated *via* activation of the Raf/ERK/MAPK pathway and results in bronchial hyperreactivity.

Increased bronchial reactivity has been reported in a group of smokers with normal pulmonary function [13]. Furthermore, there is increased bronchial hyperreactivity among children with asthma with smoking mothers [14]–[16]. An increase in bronchial hyperreactivity is a characteristic of late-phase airway reactivity [17]. Our preliminary data of sustaining SHS exposure (from acute to chronic exposure) revealed that it takes at least 8-week SHS exposure to obtain significant receptor upregulation and enhanced bronchial SMC hyperreactivity. AHR is also an important functional feature of asthmatic inflammation and chronic bronchitis. Cigarette smoke exposure induces early-stage hyperreactivity and may contribute to suboptimal lung growth during the preadolescent and adolescent years [18]. In addition, cigarette smoke causes rapid cell proliferation in the small airways and in the associated pulmonary arteries [19]. In the present study, we used a rat model to simulate passive smoking. SHS for a duration of 2 or 4 weeks did not induce bronchial hyperresponsiveness or changes in the receptor-mediated contractions in the bronchi. Eight weeks of SHS exposure resulted in enhanced expression of 5-HT_2A_, ET_B_ and ET_A_ receptors and elevated receptor-mediated contraction. The primary reasons for the reduced airflow originate in the small conducting airways [20], such as the bronchi, but not the tracheae, as previously shown in mice [5]. If the exposure continues for a longer duration, it may finally result in emphysematous destruction of gas-exchanging tissue [21].

Bronchial hyperreactivity is characterized by easily triggered bronchospasm and contraction of the bronchioles or small airways [22]. The enhanced bronchial contraction is suggested to be due to transcription and *de novo* translation of the contractile receptors [9], [23]. The increased bronchi reactivity may be derived from the upregulation of specific contractile receptors and/or the downregulation of dilator receptors in bronchial SMC [24]. Here, we have demonstrated the upregulation of the bronchial contractile 5-HT_2A_ receptors and ET receptors, while there were no changes in TP or muscarinic receptors. The receptor agonists, such as 5-HT, S6c, ET-1, U46619 and carbachol, act on their respective receptors [25] to induce bronchial contractions. We have shown that 8 weeks of SHS exposure resulted in enhanced bronchial contractile responses to 5-HT, S6c and ET-1 with increases in the values of E_max_ and/or pEC_50_, suggesting that the efficacy and/or potency of these receptors were increased.

It is well known that 5-HT receptors have many subtypes. It was reported that the 5-HT_2A_ receptor antagonist ketanserin significantly decreases ovalbumin-induced murine AHR [26]. The concentration-effect curves for 5-HT can be shifted to the right by ketanserin, indicating that the responses are mediated by 5-HT_2A_ receptors [27]. The pharmacological characterizations of the other examined receptors have been performed in previous studies by their specific receptor antagonists [8], [9], [28].

In parallel with the functional results, the mRNA and protein expression levels of the 5-HT_2A_, ET_B_ and ET_A_ receptors were increased after the SHS exposure, indicating upregulation of the 5-HT_2A_, ET_B_ and ET_A_ receptors in the bronchi. In agreement with the mRNA expression, the protein levels of the 5-HT_2A_, ET_B_ and ET_A_ receptors were increased after SHS exposure, which suggests that a transcription and translation mechanism is involved. We demonstrated an upregulation of the ET_A_ receptor protein in the smooth muscle layers of the bronchi, which is supported by a study of bronchi segments exposed to DSP [8].

The muscarinic receptors located in the smooth muscles of the blood vessels as well as in the lungs are classified as the M_3_ receptor. Activation of M_3_ receptor mediates an increase in intracellular calcium and typically causes contraction of smooth muscle such as bronchoconstriction. However, with respect to vasculature, activation of M_3_ on vascular endothelial cells also causes increased synthesis of nitric oxide, which diffuses to adjacent vascular SMCs and causes their relaxation, thereby explaining the paradoxical phenomenon on carbachol-induced tone in vessels (dilatation, with the exception of vascular endothelium disruption) and bronchi (constriction) [29]. In the present study, the contractions mediated by the M receptor agonist carbachol were not altered. Similar results have been reported for an asthma model in rats, with no significant differences in the E_max_ and pEC_50_ values of carbachol-induced bronchial contraction [30]. However, when the rats were exposed to main-stream cigarette smoke, hyperresponsiveness of the bronchial smooth muscle to acetylcholine was observed [31]. This difference may be due to the type of exposure used in these studies (active or passive). The constituents, properties and health-related effects of SHS (side-stream smoke) exposure emissions are obviously different from those of main-stream cigarette smoke [32].

Previously, we have reported that DSP *in vitro* enhanced the expression of ET_B_ and ET_A_ receptors in the bronchi [8]. The tracheal instillation of Sephadex is a fairly common method for inducing airway inflammation. This method resulted in an enhanced contractile response to S6c and ET-1 and elevated ET_B_ receptor mRNA in the bronchioles but not in the trachea [33]. It would appear that the role of upregulated ET_B_ receptors in the bronchioles was the most prominent. Furthermore, patients with asthma showed higher levels of bronchial ET_B_ receptor mRNA compared to non-obstructive subjects (mainly lung cancer) [34]. Thus, it is possible that hyperreactive lung disease is associated with altered receptor expression in bronchi; however, this hypothesis must be verified in patients. To our knowledge, there is a strong correlation between cigarette smoke exposure and inflammatory responses. Cigarette smoke caused infiltration of inflammatory cells in the tracheal SMC layer and tracheal mucous glands hypertrophy in mice [5]. Probably, these inflammatory cytokines participate to mediate the receptor regulation consequences induced by SHS exposure.

The intracellular MAPK signaling pathways are widely involved in a variety of cellular programs, including the transcription of receptors. The signaling pathways can be activated by a diverse array of stimuli, such as mitogens, osmotic stress, pro-inflammatory cytokines and growth factors [35]. The measurement of kinase phosphorylation events, such as ERK phosphorylation, may reveal new targets to modify receptor upregulation [36]. In addition, activation of MAPKs can result in mobilization of intracellular calcium thereby increasing the sensitivity of the contractile receptors [37]. In the present study, we observed that SHS exposure enhanced the phosphorylation of Raf-1 and ERK1/2, but it did not alter that of the other MAPKs, JNK or p38, indicating that SHS exposure induces Raf/ERK1/2 activation. Connected with what we have shown above, SHS exposure induced upregulation of contractile 5-HT_2A_, ET_A_ and ET_B_ receptors in rat bronchi, suggesting that the activation of the Raf/ERK1/2 pathway may be involved in the process of bronchial receptor upregulation. This hypothesis is in concert with a study reported that inhibition of ERK provides protection from the effects of acute lung injury [36]. Moreover, using other models, such as organ culture, the ERK1/2 pathway has been shown to play an important role in the ET_B_ receptor-mediated contraction in airways [27].

GW5074 is benzylidine oxindole derivative that inhibits the Raf/ERK/MAPK kinase cascade by blocking the kinase activity of Raf-1 [38]. The compound has been used in studies of the Ras/Raf-1/ERK pathway and has demonstrated the ability to inhibit polymethyl acrylate-mediated activation of ERK1/2 [39]. GW5074 has neuroprotective effect in an animal model of neurodegeneration through a Raf/ERK-related mechanism. GW5074 blocked the Raf/ERK/MAPK pathway *in vitro* with an IC_50_ of 9 nM. In cell culture, the addition of 5 μM GW5074 inhibited MAPK activation by 80%. Surprisingly, however, the treatment of cultured neurons with GW5074 also leads to Raf-1 activation [40]. The paradoxical activation is likely considered as triggering compensatory mechanisms [41]. In the present study, GW5074 was used to further demonstrate the effect of the Raf/ERK/MAPK pathway on receptor upregulation. GW5074 reduced the increased level of phosphorylated ERK1/2 protein but had no effect on Raf-1 per se. The results support our hypothesis that Raf/ERK/MAPK pathway is associated with SHS-induced bronchial receptor upregulation and this is consistent with our recent protein studies on cerebral [42] and coronary arteries [43].

Inhibition of Raf-1 by GW5074 attenuated the increased ET_A_ receptor expression in function, mRNA and protein levels, indicating that the SHS-induced upregulation occurs *via* the Raf/ERK/MAPK pathway. For 5-HT_2A_ receptors, GW5074 markedly suppressed the elevated contraction and mRNA expression but did not significantly change the protein level. The variance of sensitivity in the used methods (Western blotting is lower than real-time PCR) may account for the data inconsistency. Our results reveal at least that Raf-1 inhibitor transcriptionally reduces the SHS-induced 5-HT_2A_ receptor upregulation. In addition, GW5074 had merely a slight but not significant effect on the increased ET_B_ receptors caused by SHS. This finding suggests that Raf-1 point is not involved in the upregulation of the ET_B_ receptor in the Raf/ERK/MAPK signaling.

To summarize, we demonstrated that SHS exposure transcriptionally upregulates the contractile 5-HT_2A_, ET_B_ and ET_A_ receptors in rat bronchial SMC and causes increased bronchial reactivity. Experiments of *in vivo* treatment with Raf-1 inhibitor and Raf/ERK/MAPK phosphorylation reveal that this pathway is involved in receptor upregulation (5-HT_2A_ and ET_A_) process and bronchial hyperreactivity. The findings may provide new options for the treatment of SHS-related AHR.

## Materials and Methods

### Animals

Male Sprague-Dawley rats (weight 200–250 g) were obtained from the Animal Center of Xi'an Jiaotong University College of Medicine and maintained on normal diet, with free access to food and water. The experimental protocols for using rats were approved by the Animal Ethics Committee at Xi'an Jiaotong University. The *in vivo* exposure was performed at Xi'an Jiaotong University, China and the molecular biology experiments were done at Lund University, Sweden.

### SHS Exposure

Rats were randomly divided into 3 groups and exposed to the following conditions for 8 weeks: (a) fresh air exposure + vehicle; (b) SHS exposure + vehicle; (c) SHS exposure + Raf-1 inhibitor GW5074 (a kind gift from Prof. Yu-hai Tang, Science College of Xìan Jiaotong University, China) [38]. SHS exposure was performed in a plastic chamber (0.37 m^3^). The cigarettes were obtained from commercial brand (Marlboro, 1.0 mg of nicotine and 12 mg of tar). Two cigarettes were lit at the same time and a total of 10 cigarettes were used to be burning for 200 min per day. The rest of the time, the animals were exposed to fresh air. The detailed procedure was described previously [43]. The rats in the fresh air group were exposed to the fresh air. For treatment, the SHS-exposed rats were injected intraperitoneally (i.p.) with 0.5 mg/kg GW5074 once every day for 8 weeks. The used dosage of GW5074 was based on a previous study using an *in vivo* model [5]. The same volume of saline was administrated as sham controls. In a pilot study, the time points of 2 and 4 weeks SHS exposure were also carried out (*n* = 3–4); however, there were no significant changes in receptor-mediated contractile responses among the groups (data not shown). Therefore, these time points were not examined further. In addition, a group of rats exposed to fresh air + GW5074 (n = 8) daily for up to 8 weeks was also added. Since the results of this treatment did not differ from that of fresh air exposure + sham group, the data are not presented below.

### Bronchial Ring Segment Myograph Study

The rats were anesthetized and exsanguinated after the last day of exposure. The entire lung was removed gently and immersed in a cold bicarbonate buffer solution (mM: NaCl 119, NaHCO_3_ 15, KCl 4.6, NaH_2_PO_4_ 1.2, CaCl_2_ 1.5, MgCl_2_ 1.2 and glucose 5.5). The bronchi were then freed of adhering tissue down to the second order under a dissection microscope. The bronchi were cut into ring segments with 1–2 cm length and mounted in temperature-controlled (37°C) myograph baths (Danish Myo Technology A/S, Aarhus, Denmark) containing a bicarbonate buffer solution. A potassium-rich (63.5 mM K^+^) buffer solution was used later to test the viability of the bronchial segments and as a reference for contractile capacity. The concentration-response curves were obtained by cumulative administration of the contractile receptor agonists 5-HT, S6c, ET-1, U46619 or carbachol.

To study ET_A_ receptor-mediated contraction, the experiment began with the desensitization of the ET_B_ receptors by generating a concentration response curve for the selective ET_B_ receptor agonist S6c [28]. When the maximal contraction elicited by S6c was reached, the segments were maintained in the bath filled with the highest concentration of S6c for an additional 30 min until the contractile curves faded to a baseline level, which was considered to represent total desensitization. Then, we evaluated the ET-1 (a combined ET_A_ and ET_B_ receptor agonist)-induced concentration-contraction curve, which confirmed that the contractile response was only mediated by ET_A_ receptors. The procedure has been verified and described in a previous study [8].

### Real-time PCR

The total cellular RNA from bronchial segments was extracted using the RNeasy Mini kit following the supplier's instruction (Qiagen, Hilden, Germany). The detailed protocol was described in our previous study [23]. Specific primers were designed using the Primer Express 2.0 software (Applied Biosystems, Foster city, CA, USA) and synthesized by TAG Copenhagen A/S (Copenhagen, Denmark), or purchased from RT^2^ qPCR Primer Assay (SABiosciences, Frederick, MD, USA). The nucleotide sequences of the primers used in the investigation are shown in table 2. GAPDH which was continuously expressed at a constant level in cells was used as a reference gene.

**Table 2:** Accession numbers and primer sequence for target genes.

Gene name	Abbreviation	Accession No.	Primer sequence
Glyceraldehyde-3-phosphate dehydrogenase	GAPDH	GU214026.1	Fwd:5′-GGCCTTCCGTGTTCCTACC-3′ Rev: 5′-CGGCATGTCAGATCCACAAC -3′
5-hydroxytryptamine (serotonin) 2A receptor	5-HT_2A_	NM_017254.1	Fwd:5′-ATACCAGCATTGGCCTACAACT-3′ Rev: 5′-TAACCATGGAGCAGTCATCAAC-3′
Endothelin receptor type B	ET_B_	NM_017333.1	Fwd:5′-GATACGACAACTTCCGCTCCA-3′ Rev: 5′-GTCCACGATGAGGACAATGAG-3′
Endothelin receptor type A	ET_A_	NM_012550.2	Fwd:5′-GTCGAGAGGTGGCAAAGACC-3′ Rev: 5′-ACAGGGCGAAGATGACAACC-3′
Thromboxane A_2_ receptor	TP	NM_017054.1	Fwd:5′-ATCTCCCATCTTGCCATAGTCC-3′ Rev: 5′-CCGATGATCCTTGAGCCTAAAG-3′

### Western Blotting

Protein was extracted from the bronchial segments as previously described [9]. Briefly, after gel electrophoresis and membrane transfer, the membranes were blocked with 5% bovine serum albumin (Sigma-Aldrich, St. Louis, MO, USA) and incubated with primary antibodies (table 3) and the appropriate secondary antibodies (anti-rabbit lgG, HRP-linked antibody, #7074, 1: 2000; anti-mouse lgG, HRP-linked antibody, #7076, 1: 2000, Cell Signaling Technology, Beverly, MA, USA). Detection of protein bands was performed using Super Signal Chemiluminescent Substrate (Pierce Biotechnology, Rockford, IL, USA). Then, the membranes were developed using a Fujifilm LAS-1000 Luminescent Image Analyzer (Fujifilm, Stamford, CT, USA). β-actin was used as an internal loading control. Densitometric analysis was performed using Image Gauge Ver. 4.0 (Fujifilm).

**Table 3:** List of primary antibodies used for Western blotting.

Antigen	Abbreviation	Host	Dilution	Source
5-hydroxytryptamine (serotonin) 2A receptor	5-HT_2A_	Rabbit	1∶900	Abcam, Cambridge, UK, ab16028
Endothelin receptor type A	ET_A_	Rabbit	1∶100	Santa Cruz Biotechology, Santa Cruz, USA, sc-33536
Endothelin receptor type B	ET_B_	Rabbit	1∶500	Abcam, Cambridge, UK, ab65972
phospho-c-Raf (Ser338)	p-Raf-1	Rabbit	1∶1000	Cell Signaling Technology, Beverly, MA, USA, #9427
phospho-p44/42 MAPK (Erk1/2) (Thr202/Tyr204)	p-ERK1/2	Rabbit	1∶1000	Cell Signaling Technology, Beverly, MA, USA, #4370
phospho-SAPK/JNK (Thr183/Tyr185)	p-JNK	Rabbit	1∶1000	Cell Signaling Technology, Beverly, MA, USA, #4668
phospho-p38 MAPK (Thr180/Tyr182)	p-p38	Rabbit	1∶1000	Cell Signaling Technology, Beverly, MA, USA, #4631
c-Raf	Raf-1	Rabbit	1∶1000	Cell Signaling Technology, Beverly, MA, USA, #9422
p44/42 MAP Kinase	ERK1/2	Mouse	1∶1000	Cell Signaling Technology, Beverly, MA, USA, #4696
β-actin	β-actin	Rabbit	1∶1000	Cell Signaling Technology, Beverly, MA, USA, #4970

### Immunofluorescence

The bronchi were dissected from the lungs and fixed in 4% paraformaldehyde overnight, and then it was replaced with 0.1 M PBS. The paraformaldehyde-fixed bronchi specimens were embedded in paraffin and cut into 4-μm sections. Antigen retrieval was performed by treating the sections with 10 mM sodium citrate buffer (pH 6.0) in a microwave oven for 10 min. As described previously [9], immunofluorescence staining with primary antibody anti-ET_A_ receptors (table 3) was performed followed by a 1-h incubation with a goat anti-rabbit IgG secondary antibody conjugated with fluorescein isothiocyanate (FITC, 1∶100, Cayman Chemical, Ann Arbor, MI, USA). Then, the slides were mounted with anti-fading mounting medium containing 4′, 6-diamidino-2- phenylindole (Vectashield, Vector Laboratories Inc., Burlingame, CA, USA), which stains cell nuclei. Immunoreactivity was visualized at the appropriated wavelengths with an epifluorescence microscope (Nikon 80i, Tokyo, Japan) and photographed with an attached Nikon DS-2Mv camera.

### Statistical Analysis

All data are expressed as the mean values ± SEM, and *n* refers to the number of rats. The contractile responses to the receptor agonists in each segment are expressed in mN. Two-way analysis of variance (ANOVA) with Bonferroni's post-test was used to compare the two corresponding data points at each concentration of the two curves. The levels of mRNA for the target genes were expressed relative to the level of the housekeeping gene GAPDH. The expression levels of the target proteins were presented in relation to the level of β-actin or the amount of total protein of Raf-1 or ERK1/2. One-way ANOVA with Dunnett's post-test was used for comparison of more than two data sets. The calculations and statistical analysis were performed using Graph Pad Prism 5.0 (GraphPad Software, Inc., La Jolla, CA, USA). *P*<0.05 was considered to be statistically significant.
